# Specialized Prey Selection Behavior of Two East African Assassin Bugs, *Scipinnia repax* and *Nagusta* sp. that Prey on Social Jumping Spiders

**DOI:** 10.1673/031.010.8201

**Published:** 2010-06-29

**Authors:** Robert R. Jackson, Kathryn Salm, Ximena J. Nelson

**Affiliations:** ^1^ School of Biological Sciences, University of Canterbury, Private Bag 4800, Christchurch, New Zealand; ^2^ International Centre of Insect Physiology and Ecology (ICIPE), Thomas Odhiambo Campus, Mbita Point, Kenya; ^3^ Centre for the Integrative Study of Animal Behaviour, Macquarie University, Sydney, NSW 2109, Australia

**Keywords:** araneophagy, intraguild predation, myrmecomorphy, predatory specialization, prey-capture behavior, Reduviidae, Salticidae

## Abstract

The prey choice behavior and predatory strategies of two East African assassin bugs, *Scipinnia repax* (Stäl 1961) and *Nagusta* sp. (Hemiptera: Reduviidae), were investigated in the field and the laboratory. Both of these species are from the subfamily Harpactorinae and specialize in eating spiders. They prey especially often on social jumping spiders (Salticidae) that build nest complexes (nests connected by silk) in vegetation near the shoreline of Lake Victoria. Both reduviid species associate with these nest complexes and prey on the resident salticids. *Nagusta* sp., but not *S. repax*, form groups on nest complexes with 2–3 individuals of *Nagusta* sometimes feeding together on a single salticid. In addition to social salticids, *Nagusta* sp. preys on *Portia africana*, an araneophagic salticid that often invades the same nest complexes. *S. repax* preys on salticid eggs and also on *Nagusta*. Although they avoid ants, *Nagusta* and especially *S. repax* prey on ant-mimicking salticids, suggesting that sensory modalities other than vision play a dominant role in prey detection.

## Introduction

The mouthparts of true bugs (Hemiptera, suborder Heteroptera) are structures specialized for piercing and sucking, and many of these insects are primarily phytophagous (Goodchild 1966; Miles 1972; Cobben 1978). However, trophic switching has been a common theme in heteropteran evolution (Cohen 1996) with hematophagous (Lehane 2005) and predatory species (Schuh and Slater 1995) illustrating that heteropteran mouthparts can also be effective at piercing animal tissue. Both of these departures from phyotophagy can be found in the family Reduviidae. Triatominine reduviids are hematophagous (Gurtler et al. 1997; Sandoval et al. 2000; Schaefer 2005; Tartarotti et al. 2006), but it is probably accurate to characterize the majority of the species in this large family (more than 6,600 described species, Weirauch 2008) as predators that feed primarily on other arthropods. However, judging from the literature and from the new information reported here, it would be misleading to characterize reduviids as “generalized” predators.

This study considers the predatory strategies of two East African reduviids, *Nagusta* sp. indet. (hereafter referred to as *Nagusta*) and *Scipinnia repax* (Stäl 1961) and provides evidence that these reduviids are araneophagic (i.e., that they specialize in preying on spiders), with their prey often being jumping spiders (Salticidae). Salticidae is an unusual spider family because, although vision is poorly developed in most spiders, they have complex eyes and exceptional eyesight (Land 1969; Blest et al. 1990; Harland and Jackson 2004). Most salticids are solitary hunters that spend their lives outside webs and prey primarily on insects (Richman and Jackson 1992), but the biology of *Nagusta* and *S. repax* appears to intersect strongly with three atypical minorities in this large spider family that includes more than 5,000 species in over 500 genera (Platnick 2008): social salticids, araneophagic salticids, and myrmecomorphic salticids (i.e., salticids that are ant-like in appearance).

## Materials and Methods

### General

The study site was the Thomas Odhiambo Campus of the International Centre for Insect Physiology and Ecology (ICIPE) in the town of Mbita Point, next to Lake Victoria in western Kenya (0° 25′ S to 0° 30′ S by 34° 10′ E to 35° 15′ E, 1200 MASL, mean annual temperature of 27° C). In this habitat, non-biting midges (Diptera: Chironomidae & Chaoboridae), known locally as ‘lake flies’, are exceedingly abundant (Beadle 1981) and support enormous populations of salticids (Jackson 1999). The orb webs of *Tetragnatha, Nephila*, and *Nephilengys* and the dome webs of *Cyrtophora* were especially common in the study sites. The webs of *Argyrodes* were enmeshed and difficult to discern within the webs of these larger spiders (see Whitehouse 1986). The individual webs of all of these species tended to run together, forming large interspecific web complexes in the vegetation.

Salticids typically build cocoon-like silk nests in which they take shelter, molt, mate, and oviposit, and salticid nests are usually isolated. However, a minority of the species in the family Salticidae live in nest complexes where individual nests are interconnected by silk (Jackson 1986a, b; 1999). In the study site, nest complexes were common in the vegetation and were often surrounded by, if not touching, the web complexes. There were three social salticids with which *Nagusta* and *S. repax* often associated: *Menemerus* sp. indet. (hereafter referred to as *Menemerus*) and two undescribed species of *Pseudicius* (*Pseudicius* species A and *Pseudicius* species B). Other salticids of importance for this study were: *Portia africana* (Simon 1885), an araneophagic salticid; *Myrmarachne* sp. indet. (hereafter referred to as *Myrmarachne*), a myrmecomorphic species; and *Evarcha culicivora* (Wesolowska and Jackson 2003), a salticid that feeds indirectly on vertebrate blood by choosing blood-carrying mosquitoes as their preferred prey (Jackson et al. 2005, Nelson and Jackson 2006a) and a few species that could not be identified to genus.

### Group composition and location of *Nagusta* and *S. repax* in the field

A survey was carried out over six successive days in January 2002 in an area where *Nagusta* and *S. repax* were known to be common from previous observations. However, reduviids or spiders were not collected from this area during the previous 12 months. Dominant trees in the survey site were *Citrus* spp.; mango, *Mangifera indica* L.; kapok, *Ceiba pentandra* L.; and fig, *Ficus benjamina* L. During the survey, we examined all leaves that could be reached without a ladder and recorded the location of each individual *Nagusta* and *S. repax* found.

During casual observations, a few individuals of *Nagusta* and *S. repax* were seen on tree trunks or the walls of buildings standing on or near spider silk, but it was clear that the majority were on the leaves of trees and shrubs and were associated with salticid nest complexes. This was the rationale for including only tree and shrub leaves in the survey.

‘Salticid silk’ was used as a collective term for solitary salticid nests and salticid nest complexes, and the term ‘webbing’ was used for egg sacs and disused web silk. ‘Associated with webbing’ and ‘associated with salticid silk’ were terms for instances of an individual of *S. repax* or of *Nagusta* being either on (i.e., touching) or close to (i.e., within 10 mm of, but not on) webbing or salticid silk, respectively. ‘Associated with spider silk’ was a collective term for any instance of an individual of *S. repax* or *Nagusta* being on or close to either webbing or salticid silk. The term ‘group’ was used for instances of finding two or more reduviid individuals associated with the same salticid silk or, if not associated with salticid silk, within 10 mm of each other. When there was only one reduviid associated with salticid silk, it was recorded whether it was on or only near nest silk. A ‘sighting’ refers to an individual or a group of reduviids that was found at a single location (solitary salticid nest, salticid nest complex, webbing, or a site separated from spider silk). Data for numbers of reduviid individuals are also presented. However, disturbance caused by sampling ruled out reliable judging of whether individuals in groups had been on or only near nest silk. Although the data presented here came from the survey only, these data were consistent with extensive casual observation over a 10-year period.

### Prey and predatory behavior of *Nagusta* and *S. repax* in the field

Prey records were obtained opportunistically during the course of casual observation in the field (1998 to 2008). This was achieved by collecting the predator and the prey for identification whenever we found individuals of *Nagusta* or *S. repax* feeding. On a casual basis, we occasionally made opportunistic observations (5–60 min in duration) of reduviid predatory sequences.

Based on prey-body length relative to predator-body length, four prey-size categories were defined: small, the prey's body length less than 0.1 times the predator's body length; medium, 0.1 to 0.5 times the predator's body length; large, 0.5 to 1.0 times the predator's body length; very large, more than 1.0 times the predator's body length. ‘Larger’ was a collective term for all prey larger than ‘small’. The term “hatchling” was used for spiders that had recently emerged from eggs (pale coloration; body length *c.* 1 mm) and “juvenile” was used for immature spiders and reduviids that were 2+ mm in body length.

*A posteriori* exact logistic regression tests comparing prey of *Nagusta* and *S. repax* were performed for each of the two factors (prey size and prey family), adjusting for the other factor. Statistics were performed using LogXact 8 (Cytel Inc., Cambridge, MA, 2007), with 100,000 Monte Carlo samples used to estimate probability, χ^2^ tests of independence were performed using SPSS v. 16.

### Predatory behavior of *Nagusta* and *S. repax* in the laboratory

For maintenance cages and for test arenas, Petri dishes (inner diameter = 90 mm) were turned upside down so that the slightly narrower side, which normally would be on the bottom, was on the top (called the “lid” from here on). The slightly wider part, normally lying under the lid, was used as the “base.” The lid was removed and a green leaf that was wider than the dish was pressed into the base so that it fit snugly against the bottom of the dish. When the lid was replaced, the perimeter of the leaf extended to the outside of the dish, ensuring that there was no space through which predators and potential prey inside dishes could move under the leaves.

The leaves used were from yam-bean plants (*Pachyrhizus ahipa*); these were particularly suitable because they were wider than the Petri dishes, highly pliable, and resistant to being torn. We chose this cage design, including the leaf, after trying numerous alternatives including the bare plastic cages that have been standard in research on jumping spiders (see Jackson and Hallas 1986). The rationale for the leaf was that *S. repax*, and especially *Nagusta*, seemed considerably more responsive to prey when they were standing on a leaf.

A damp cotton roll (diameter = 10 mm; length = 20 mm) was kept centered on the top of the leaf. When the leaf began to turn brown it was replaced, but the leaves usually stayed green for four to seven days. For routine maintenance, prey was added to the dish three times per week (Monday, Wednesday, and Friday) in sufficient numbers so that the reduviid could feed to satiation. Midges, which were used as prey, were collected as needed, and hatchlings of *Evarcha culicivora*, also used as prey, were acquired from laboratory culture. Encounters were staged between the reduviids and ants (*Camponotus* sp. and *Crematogaster* sp.). The reduviid normally spent most of its time standing on the leaf, and by carefully lifting the lid, potential prey could be introduced into the dish without causing noticeable disturbance.

Prior to testing, hunger level was standardized by keeping each reduviid (‘test bug’) in a clean Petri dish without prey for five days. Three testing procedures were adopted: (1) no-silk tests, (2) prey-and-silk tests, and (3) predator-on-silk tests. No-silk tests were staged by placing the potential prey directly onto the yam-bean leaf, but prey-and-silk and predator-on-silk tests were staged by placing a leaf or a cut piece of a leaf (length *c*. 30 mm, width *c*. 20 mm; hereafter referred to as ‘small leaf’) on the large yam-bean leaf. The small leaves, which came from *Citrus, Ficus benjamina*, or *Mangifera indica*, were collected immediately prior to prey-and-silk testing and one day before predator-on-silk testing. When collected, there was always webbing, a salticid nest complex, or a solitary salticid nest on the small leaf. Immediately before testing began, the cotton roll was removed from the Petri dish. A prerequisite for continuing was that the predator inside the dish was standing on the yam-bean leaf (no-silk and prey-and-silk tests), or on the small leaf (predator-on-silk tests). This prerequisite was almost always met.

Testing began by placing the prey (no-silk tests), or by placing a small leaf on top of the yam-bean leaf and then replacing the lid. Once testing began, observation of the test bug was continual for the next 60 min, or until predation occurred. For prey-and-silk tests the prey were salticids in solitary nests, salticids in nest complexes, or *Tetragnatha* on webbing (the spiders were always hatchlings or juveniles). For some prey-and-silk tests, *S. repax* was the predator and the prey was an individual of *Nagusta* standing on a solitary salticid nest or a nest complex. For predator-on-silk tests, all resident arthropods were removed from the small leaf and silk before placing the leaf in the Petri dish with the predator.

When describing the reduviid's predatory behavior, terms and conventions from earlier studies were used (see Jackson and Hallas 1986), and the necessary adjustments were made for referring to an insect instead of a spider. Frequencies of occurrence: ‘usually’, ‘often’, and ‘typically’ indicated *c*. 80% or more; ‘sometimes’ and ‘occasionally’ indicated 20–80%; ‘infrequently’, ‘rarely’ and ‘on rare occasions’ indicated 20% or less. Legs I, II, and III referred to the reduviid's anterior, middle, and posterior pair of legs, respectively. *Attack* referred to the insertion of proboscis into prey. *Lunge* described the reduviid extending legs III and moving its body rapidly forward, forcefully contacting the prey. *Quiescent* referred to prey staying stationary and inactive, in its normal rest posture. *Quiet* referred to prey being stationary, but not entirely inactive (i.e., slowly turning about, grooming, or repositioning legs).

A bout of ‘antennating’ was a period of continuous up-and-down motion of an antenna, which sometimes included numerous complete cycles from the most dorsal to the most ventral position and back. For antennating, amplitude was the distance between the extreme positions in a movement sequence (i.e., the distance between the most dorsal and the most ventral position). Three terms were used for phasing. *Matching* referred to both antennae moving to their most dorsal positions simultaneously and to their most ventral positions simultaneously. *Alternating phasing* referred to situations in which one antenna was in its most dorsal position and the other was in its most ventral position (phase difference 180°). *Irregular phasing* referred to any position between matching and alternating.

## Results

### Group composition and location of *Nagusta* and *S. repax* in the field

For full data on the number of individuals and the number of sightings when more than one individual was found together, refer to Tables 1 and 2. Almost half of the individuals of *Nagusta* found were juveniles (47.7%). Of 180 adults, 47.2% were females and 52.8% were males. During casual observations, groups of a dozen or more *Nagusta* were sometimes seen, although groups of two to five were more typical. The majority (83.7%) of *Nagusta* individuals were found in groups, and this was true both for adults (78.9%) and juveniles (89.0%). Of the group sightings, the most common group composition was of two individuals (55.3%), with successively fewer sightings for larger groups: group size of three (19.4%), of four (15.5%), and of five (9.7%).

For *S. repax*, there were 62 sightings, and as there were no instances of more than one *S. repax* individual in a group, the number of individuals was the same as the number of sightings. This included 19 instances in which *S. repax* was associated with a nest complex in the company of one (73.7%) or two (26.3%) *Nagusta* individuals. About half of the *S. repax* were juveniles (42.0%), with 61.1% of the 36 adults being females and 38.9% being males.

**Table 1.**
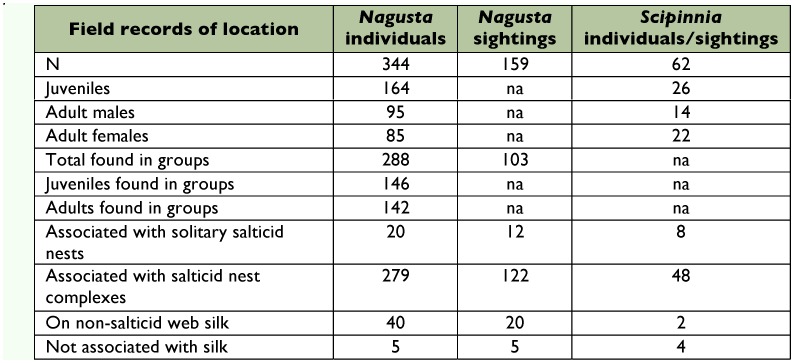
Location and sex/age grouping of *Nagusta* sp. (counted individually and as ‘sightings’ when forming part of a group) and *Scipinnia repax* in the field.

**Table 2.**
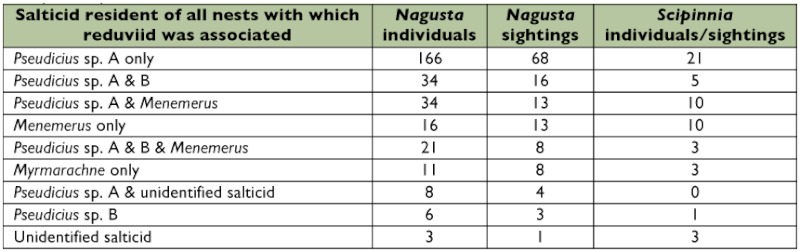
Residency of salticid nests with which *Nagusta* sp. (counted individually and as ‘sightings’ when forming part of a group) and *Scipinnia repax* were found in the field.

**Figure 1.**
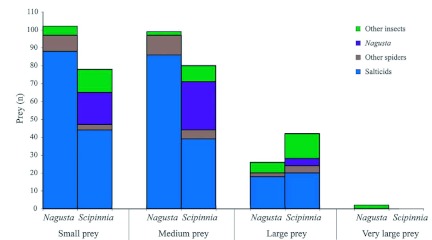
Prey of *Nagusta* sp. and *Scipinnia repax* in the field. See text for definitions of prey sizes. High quality figures are available online.

Both species were often associated with spider silk (*Nagusta*: 98.5%, *S. repax*: 93.5%) and especially with salticid silk (*Nagusta*: 86.9%, *S. repax*: 90.3%). Of those associated with salticid silk, the majority (*Nagusta*: 93.3%, *S. repax*: 85.7%) were associated with nest complexes instead of solitary nests (Figure 1).

Based on the identity of the resident salticids, there were nine categories of solitary nests and nest complexes with which the reduviids associated (Table 2). There were clear differences in *Nagusta* numbers (*n* = 299) found with different salticid species when these were all counted, regardless of the presence of other salticid species. Ranked, these were: *Pseudicius* sp. A, 88.0%; *Menemerus*, 23.7%; *Pseudicius* sp. B, 20.4%; *Myrmarachne*, 3.7%; salticids that could not be identified to genus, 3.7%. The sum of these percentages exceeds 100 because any given salticid in a complex can contribute to more than one data point. For the 56 *S. repax* individuals associated with salticid silk, 37.5% were associated with *Pseudicius* sp. A only, 17.9% with *Menemerus* only, 17.9% with *Pseudicius* sp. A and *Menemerus*, 8.9% with *Pseudicius* sp. A and B, 5.4% with *Myrmarachne* only, 5.4% with a salticid that could not be identified to genus, 5.4% with *Pseudicius* sp. A and B and *Menemerus*, and 1.8% with *Pseudicius* sp. B only.

### Prey and predatory behavior of *Nagusta* and *S. repax* in the field

Most (79.1%) of the *Nagusta* individuals (*n* = 229) were found feeding on a single prey item. When two or more individuals were feeding together on the same prey item (20.9%)), it was referred to as ‘group feeding’. Feeding groups were comprised of two individuals (68.2% of the 85 individuals found feeding in a group) or three individuals (31.8%).

**Figure 2.**
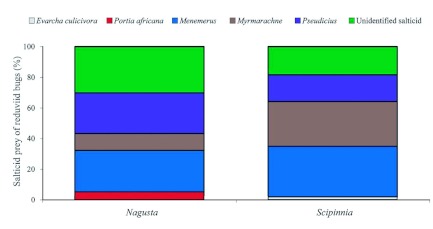
Salticid prey of *Nagusta* sp. (*n* = 192) and *Scipinnia repax* (*n* = 103) in the field. High quality figures are available online.

*Nagusta* typically fed on small (44.5%) or medium (43.2%) prey items, with large (11.4%) and very large (0.9%) prey accounting for less than a quarter of the observations (Figure 2). *S. repax* prey (*n* = 200) also tended to be small (39.0%) or medium (40.0%), but large prey (21.0%) were more frequent than in *Nagusta*'s diet. When feeding alone, *Nagusta* usually (66.0%) fed on small prey items (medium: 28.5%, large: 4.9%, very large: 0.7%). However, when group feeding, only 8.2% of the prey were small, 68.2% were medium, 22.4% were large, and 1.2% were very large. In all instances of *Nagusta* feeding in a group of three, the prey was a medium or large salticid. Considering all instances of *Nagusta* feeding in a group, 90.6% of the individuals were feeding on salticids, whereas 80.0% of the *Nagusta* individuals feeding alone were feeding on salticids.

Salticids formed the vast majority of the prey of *Nagusta* (83.8%), followed by non-salticid spiders (9.6%) and insects (6.6%). In *S. repax*, salticids accounted for rather less (51.5%), while insects accounted for more (40.0%) of the prey. Non-salticid spiders were preyed in similar proportions to *Nagusta* (6.0%), but *Spininnia* also preyed on salticid eggs (2.5%).

**Figure 3.**
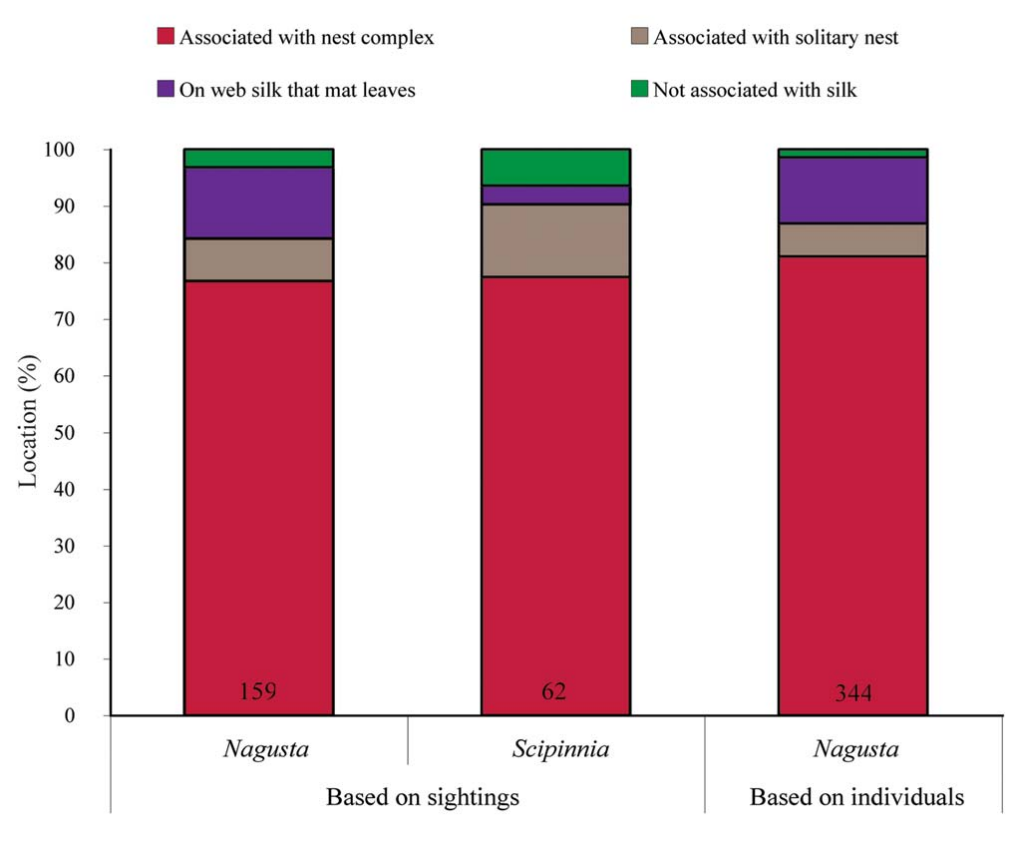
Comparison of location records for *Nagusta* sp. and *Scipinnia repax* (*n* within each bar); (The number of sightings and the number of individuals was the same for *S. repax*, but not for *Nagusta* because *Nagusta* often was in groups). High quality figures are available online.

Of the 192 salticids in the *Nagusta* prey records, 30.2% could not be identified to genus (Figure 3). Identified salticid prey were, from most frequent to least frequent: *Menemerus, Pseudicius* sp. A, *Myrmarachne*, and *Portia africana* (Figure 3). Of the 103 salticids on which *S. repax* was feeding (Figure 3), most *were Menemerus*, followed by *Myrmarachne, Pseudicius*, and *Evarcha culicivora* (18.4% could not be identified to genus). *S. repax*, when feeding on small salticids, sometimes (14.6%) had its proboscis extended through nest silk and into a prey.

**Table 3.**
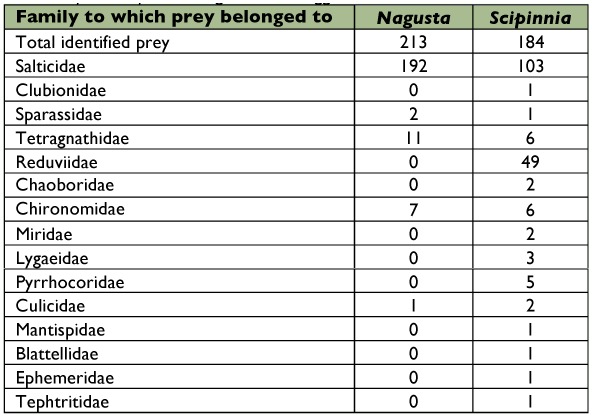
Assassin bug prey. Each prey was counted only once, regardless of whether it was being fed on by multiple bugs. Includes data only for prey that could be identified to family. Does not include 5 instances of *Scipinnia repax* feeding of salticid eggs.

Nine of the 22 non-salticid spiders from *Nagusta* prey records could not be identified to family (40.9%). The identified non-salticid prey were *Tetragnatha* (50.0%) and sparassids (9.1%). The 12 non-salticid spider prey of *S. repax* were *Tetragnatha* (50.0%), a clubionid (8.3%), a sparassid (8.3%), and spiders that could not be identified to family (33.3%).

Seven of the 15 insects being fed on by *Nagusta* could not be identified to order (46.7%). The identified insects were all dipterans: chironomid midges (46.7%) and culicid mosquitoes (6.7%), (Table 3). In contrast, heteropterans were especially common in the 80 records of *S. repax* feeding on insects (77.5%), and *Nagusta* was the most common of *S. repax*'s heteropteran prey (79.0% of the 62 heteropterans; 24.5% of all prey records). The other heteropterans were *Dysdercus, Nysius*, mirids, and various bugs that could not be identified to family. *S. repax*'s dipteran prey were, from most to least frequent: Chironomids, chaoborids, culicids, and tephritids. Other prey included three insects that could not be identified to order: a caterpillar, a cockroach, a mayfly, and a mantispid (Table 3).

### Demographics and diet of *S. repax* and *Nagusta*

**Figure 4.**
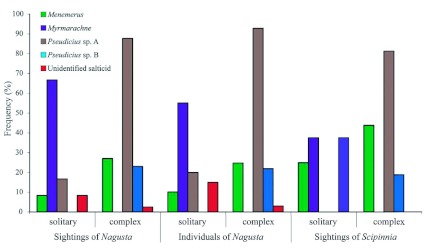
Associations of reduviids and different salticid species (expressed as percentages) in solitary nests and in nest complexes. Based on number of sightings and numbers of individuals (these are identical for *Scipinnia repax*). Categories labeled ‘nest complex’ exceed 100% because when there are salticids belonging to different species in same nest complex, each species contributes to count for the individual reduviid. High quality figures are available online.

The demographics of the two species were similar in most respects: 24.7%, 27.6%, and 47.7% of the 344 *Nagusta* and 35.5%, 22.0%, and 41.0% of the 62 *S. repax* were females, males, and juveniles, respectively. Additionally, whether or not reduviids were not associated with silk, and if they were, whether they were associated with webbing or with salticid silk (complexes or solitary nests) was similar for the two reduviid species (Figure 1). For both *S. repax* and *Nagusta, Pseudicius* sp. A was the salticid species in nest complexes with which both reduviid species most often associated, and *Myrmarachne* was the salticid in solitary nests with which both reduviid species most often associated (Figure 4).

However, there were some interesting differences between the two reduviid species. *Nagusta*, but not *S. repax*, was often found in groups and more than one individual often shared the same prey. There was a significant difference between *Nagusta* and *S. repax* regarding the size of prey they were found with (the single case of ‘very large’ prey was classified as ‘large’ prey for ease of analysis), once adjusted for prey type (exact score 8.232, p = 0.016). Significantly more *S. repax* than *Nagusta* took large prey compared with small prey (p = 0.042, Odds Ratio 2.195) and took significantly less medium prey compared with large prey (p = 0.008, Odds Ratio 2.780), but there was no difference in the likelihood of either predator to attack medium or small prey (p = 0.513, Odds Ratio 1.235) (Figure 2).

There was also a significant difference in the distribution of prey family for each predator, when adjusted for prey size (exact score 96.57, < 0.0001). However, LogXact was unable to perform tests (adjusted for prey size) on these differences due the complexity of the calculations. Instead, χ^2^ tests of independence were used to compare the caught prey between the two species (Figure 2), not adjusted for prey size. Both reduviid species preyed on salticids more often than any other prey category, but the extent to which salticids dominated the prey records was greater for *Nagusta* than for *S. repax* (χ^2^ = 51.99, p < 0.001). The remainder of *S. repax*'s prey was primarily insects, and particularly *Nagusta* species (Figure 2).

In general, the different salticid species present in the prey records were similar in proportion for the two reduviid species (Figure 3). However, *Myrmarachne* was an exception, as it was found in *S. repax*'s prey records more often than in *Nagusta*'s (χ^2^ = 15.51, < 0.001).

When seen feeding on salticid eggs and salticid hatchlings that were in nests in the field, *S. repax* was standing on or beside the nest with its proboscis extending through the silk and into an egg or a hatchling. In these instances, a living adult female salticid was usually inside the nest and standing over, or at least near, the egg mass. Despite *S. repax*'s proboscis being nearby, the salticid remained quiescent. On three occasions, a dead and desiccated adult salticid female was in the nest next to the eggs on which *S. repax* fed, suggesting that the reduviid may have fed on the adult salticid.

### Predatory behavior of *Nagusta* and *S. repax* in the laboratory

Laboratory results confirmed that both reduviid species attacked, killed, and fed on representatives of each category of prey identified from the field records. Encounters between reduviids and ants confirmed that ants were avoided; yet both readily preyed on *Myrmarachne* (see above), the myrmecomorphic salticid.

Both reduviids tended to move slowly, but *Nagusta*'s disposition appeared distinctively more sluggish than *S. repax*'s. Even when poked with a finger or a pair of forceps, *Nagusta* rarely moved fast. Usually the first noticeable reaction by *Nagusta* or *S. repax* to potential prey was only after the prey made head-on contact with, or at least came to within 1–2 mm, of the reduviid (usually only when approached head-on, although there were rare instances of the reduviid turning as much as 180° to face nearby prey in other orientations). There were rare instances of the reduviid turning and facing active prey that was two to four body lengths away, raising its body, partially extending its proboscis, and walking toward the prey.

*Nagusta* and *S. repax* usually attacked prey by making a sudden lunge during which its legs I went over the prey, its body moved downward, and its proboscis was inserted. On rare occasions, when the prey was small and quiet, or quiescent, the reduviid omitted the lunge and attacked simply by slowly inserting its proboscis. Soon after attacking, *S. repax* usually moved its legs away, raised its body, and held on to the prey with only its proboscis. At the end of a lunging attack, *Nagusta* was usually in a distinctive posture in which it appeared to be flattened against the substrate (legs I, and sometimes II, highly flexed and lying on or close to the substrate, with tarsi on the prey close to the insertion point of the proboscis; body resting on the substrate). This posture was sometimes maintained for 60 s or longer, but eventually *Nagusta* moved its legs away and raised its body, with its proboscis still secured to the prey.

Before they attacked, *Nagusta* and *S. repax* usually antennated their prey (i.e., *Nagusta* or *S. repax* moved its two antennae up and down, lightly touching the prey on the down stroke). Antennating was characterized by highly variable phasing of movement by the two antennae, with frequent switching occurring between matching, alternating, and irregular; not only between bouts, but also within single bouts. Typical amplitude, rate, and bout length were 2–3 mm, 2 strokes s^-1^, and 1–2 s, but this also varied considerably. Antennating was especially pronounced when the prey was a salticid, and salticids rarely fled when antennated by a reduviid. Salticids that were walking while being antennated sometimes continued to walk without noticeably changing their gait, and it was typical for salticids that were quiescent when the reduviid began to antennate to remain so or pivot about while standing in place.

When prey was quiet or quiescent, the reduviid sometimes stopped antennating and rested its antennae on the prey, sometimes with its legs I raised and arched out. Sometimes the reduviid also rested its extended proboscis on the prey's body. If the prey remained quiet or quiescent, the reduviid sometimes kept its antennae (or its antennae and its proboscis) resting on the prey for 60 s or longer, and then attacked. However, if the prey became more active, the reduviid usually renewed antennating, often with the prey then calming down.

When directed at larger prey, attacks were sometimes unsuccessful because the reduviid's proboscis failed to hold the prey, and the prey moved away. After unsuccessful attacks, prey usually moved away and the reduviids rarely followed prey that moved rapidly. However, when the prey moved away slowly *S. repax* often, but *Nagusta* only rarely, followed. While following the prey, *S. repax* sometimes kept its body elevated and its proboscis extended all the while adopting a choppy gait in which it took only a few steps at a time between pauses that lasted about 1 s each. Should it fail to catch up with the prey within about 60 s, *S. repax* usually desisted. If it did get close, *S. repax* usually attacked again. Should the second attack be unsuccessful, *S. repax* usually desisted. If the prey being followed became quiescent, *S. repax* sometimes moved around so that it approached the prey from behind. While close behind slowly-walking prey, *S. repax* sometimes antennated and repeatedly extended its proboscis, often with its body elevated. Sometimes *S. repax* eventually succeeded at inserting its proboscis into the moving prey's body.

*Myrmarachne* resembles ants not only in static appearance, but also in adoption of a continual zigzag style of locomotion similar to an ant's, instead of the stop-and-go gait that is characteristic of most salticids (Richman and Jackson 1992). When antennated and probed by a reduviid, there was usually little or no noticeable change in *Myrmarachne*'s locomotion, and the reduviid rarely managed to mount an attack before *Myrmarachne* had moved away. *S. repax* rarely followed *Myrmarachne* after an unsuccessful attack.

With the prey being typically quiescent and head on when attacked, the initial penetration of the prey by the reduviid's proboscis was usually in the head of an insect or the cephalothorax of a salticid. With the exception of predation by *S. repax* on *Nagusta*, the reduviids appeared reluctant to attack larger prey. *S. repax*'s encounters with *Nagusta* were remarkably similar to *S. repax*'s encounters with salticids. *Nagusta* usually showed little sign of alarm when contacted and antennated by *S. repax. S. repax* was especially inclined to make its initial attack in *Nagusta*'s head, sometimes walking over *Nagusta* from the rear or the side and postponing an attack until positioned with its proboscis over *Nagusta*'s head. Even in these instances, *Nagusta* usually displayed little sign of alarm.

When initial proboscis insertion was located somewhere other than the head or cephalothorax of the prey, the reduviid usually moved to the prey's head or cephalothorax within about 60 s after the prey became quiescent. It was usual for small prey to become quiescent almost immediately, regardless of where the predator made its first insertion. Larger prey also became quiescent within a few seconds when insertion was in the prey's head or cephalothorax. However, when initial insertion was in a larger prey's abdomen or a leg, complete quiescence sometimes took longer than 10 min. The predator's proboscis usually impaled the dorsal side of stationary prey. However, if prey was walking when attacked by *S. repax*, it sometimes seemed to get knocked about and ended up being impaled through its side or from underneath.

After feeding for several minutes from the prey's head or cephalothorax, the reduviid usually began bouts of frequent repositioning of its proboscis on the prey's body. *Nagusta* only rarely fed from appendages, but *S. repax* often ended feeding bouts by concentrating on the antennae and the legs of insects, or the legs and palps of spiders.

It was typical for *Nagusta* to remain quiescent whenever a salticid was actively walking about, with the salticid sometimes touching or even walking on top of *Nagusta*, either without provoking an overt reaction, or at most stimulating *Nagusta* to adjust its posture or to step aside. Later, when the same salticid was walking slowly, the quiescent *Nagusta* sometimes captured it. Typical sequences in which *Nagusta* captured salticids began with *Nagusta* standing quiescent near, and facing toward, a salticid's nest door and suddenly lunging at the salticid as it left its nest. There were also rare instances when *Nagusta* faced away from a nest and attacked a salticid that was approaching the nest, and when *Nagusta* attacked small salticids that were walking about on salticid silk, on webbing, or not on spider silk at all. *Nagusta* especially readily preyed on hatchlings in the laboratory and the small salticids on which we saw *Nagusta* feeding in the field appeared often to be hatchlings, but this was difficult to discern from an already fed-upon prey item. *Nagusta* would not eat salticid eggs by penetrating salticid silk, but it readily ate eggs that were exposed when the silk had been removed.

When attacked, salticids sometimes pulled away before *S. repax* fully inserted its proboscis, but even in these instances the salticid showed little sign of alarm and, instead, usually walked away in its normal gate. Larger salticids sometimes walked away with *S. repax*'s proboscis securely inserted, dragging *S. repax* along, often with *S. repax* rolling over on its side or its back. While being dragged, *S. repax*'s proboscis sometimes came loose after a few seconds, whereupon the salticid walked away and appeared unharmed. However, if the *S. repax* kept its proboscis in place, the salticid eventually succumbed and *S. repax* fed.

When encounters were staged in the laboratory between *S. repax* and salticid nests (or nest complexes) inside which there were eggs or hatchlings (and occasionally adult females), *S. repax* walked slowly onto the nest and then became quiescent for a period lasting from a few seconds to several hours. Eventually, *S. repax* began changing its position on the nest by intermittently and slowly stepping or pivoting about, pushing its proboscis slowly through the silk and slowly probing within the nest until contacting a hatchling or an egg and inserting its proboscis into it with a sudden, forceful thrust downward. If it failed to impale an egg or hatchling, it removed its proboscis and another quiescent period usually followed, after which *S. repax* inserted its proboscis again in another location. Once impaled, salticid hatchlings usually became completely quiescent almost immediately. In contrast to when prey was outside the nests, proboscis insertion when a hatchling was inside a nest was not restricted primarily to the cephalothorax, and it was uncommon for *S. repax* to shift the position of its proboscis within prey. Sometimes *S. repax*'s proboscis touched the adult salticid's body or one of its legs, with the salticid remaining quiescent or simply stepping aside. Although *S. repax* seemed hesitant to attack the adult salticid, there were rare instances of *S. repax* lunging down in an apparent attempt to penetrate the adult with its proboscis. However, the salticid always moved away and did not appear especially alarmed.

## Discussion

The extensive field data from this study, together with laboratory findings, suggest that *S. repax* and *Nagusta* are specialized predators that target spiders as prey (araneophagy), and target salticid spiders in particular. A close look at the literature suggests that the evolution of specialized predatory strategies may have been especially common in the Reduviidae. Obligate predatory specialization on millipedes may have evolved in the Ectrichodiinae (Miller 1953; Louis 1974) and there are species in the subfamily Salyavatinae that use special predatory tactics for targeting termites as preferred prey (Miller 1971; McMahan 1982, 1983a, 1983b). Termite-eating species are also found in the Acanthaspinae (Odhiambo 1958), but myrmecophagy (specialized predation on ants), rather than termitophagy, appears to be dominant in this reduviid subfamily (Brandt and Mahsberg 2002; Jackson and Pollard 2007). Holoptilinae is another subfamily in which myrmecophagy may be both common (Jacobson 1911; Weirauch and Cassis 2006) and ancient (Poinar 1991, 1993). Harpactorinae, the subfamily to which *Nagusta* and *S. repax* belong, is also known for possible examples of predatory specialization. Some harpactorines prey especially on bees (da Silva and Santana 2004), and others appear to target phytophagous heteropterans, especially genus *Dysdercus* (Kirkpatrick 1957; RRJ unpubl.).

*Nagusta* and *S. repax* are the first harpactorines for which araneophagy has been documented, but araneophagy may be common in the reduviid subfamily, Emesinae. Reports on species from various emesine genera (Howard 1901; Smith 1910; Wickham 1910; Readio 1927; Dicker 1941; Usinger 1941; Brown and Lollis 1963; Wygodzinsky 1966; Cobben 1978; RRJ unpubl.), but especially *Stenolemus* (Hickman 1969; Snoddy et al. 1976; Hodge 1984), suggest web-building spiders are routine prey of many emesines. The most thorough study of emesine predatory behaviour has been on *Stenolemus bituberus*, a species that manipulates web silk with its appendages, thereby making signals with which it controls the behavior of the resident spider (Wignall and Taylor 2008).

*Nagusta* and *S. repax*'s style of araneophagy differs from the web-invading style of *Stenolemus*. Although we observed *Nagusta* and *S. repax* preying on web-building spiders, this was on the disused webbing that matted vegetation, not in the spiders' functioning prey-capture webs. For these two harpactorines, the dominant spiders in prey records were salticids. Only a few salticid species build webs and with one exception, the salticids eaten by *Nagusta* and *S. repax* were cursorial salticids, not web builders. The exception was *Portia africana*, a species belonging to a genus of salticids known for building prey-capture webs and for being web-invading araneophagic predators (Harland and Jackson 2004). However, no evidence of the reduviids preying on *P. africana* in webs was found. Instead, when predation on *P. africana* was observed, it was always in a nest complex, suggesting the possibility that the nest complexes of social salticids set the stage for complex predatorprey relationships that include araneophagic predators preying on other araneophagic predators. *Nagusta* appears to take advantage of opportunities to prey on *P. africana* when both *Nagusta* and *P. africana* make predatory forays into the same salticid nest complexes (Jackson et al. 2008). *S. repax* also appears to exploit opportunities to prey on an araneophagic predator that visits salticid nest complexes. However, in *S. repax*'s case, the araneophagic prey is *Nagusta* instead of *P. africana*.

Although ambushing appears to be the basic predatory tactic of both *Nagusta* and *S. repax*, the predatory strategies of these two reduviid species differ in their details. *Nagusta* seemed to prey on salticids primarily by ambushing them as they left their nests and was, compared with *S. repax*, less prone to pursue prey that moved away. Both reduviids preyed primarily on salticids that were small, but *S. repax* appeared to be somewhat more inclined than *Nagusta* to take somewhat larger salticids. *S. repax* also preyed often on *Nagusta*.

Intraguild predators are those that both prey on each other and compete for the same prey (Polis 1991). Here we provide evidence that *S. repax* and *Nagusta* are intraguild predators. Intraguild predation has recently been extensively investigated because, as the predators feed at more than one trophic level, there are profound implications for food web dynamics. It is now clear that trophic cascades are affected in ways far more complex than previously appreciated (e.g., Moya-Larano and Wise 2007). Intraguild predation has ecological implications for the co-occurrence of these species. While this was beyond the scope of this study, it is interesting to note that *Nagusta* was found roughly five times as often as its predator, *S. repax*. This distribution may be driven by the possibility that *S. repax* is a predator at a higher trophic level than *Nagusta*.

*Nagusta* and *S. repax* routinely antennated prey before attacking and when antennated, salticids showed surprisingly little sign of being alarmed. It was also interesting that, when antennated by *S. repax, Nagusta* showed little sign of being alarmed. On the contrary, prey seemed to become calmer when antennated. The characteristics of antennating that are responsible for a calming effect on prey are not known, but it may be that phasing is especially important. Continually shifting phase relationships between the two antennae may give tactile signals that, when received by the prey, have no clearly discernible pattern and are not being readily identified as coming from a predator. Something similar has been suggested for *Portia*'s behavior of making signals by silk-line manipulation. When making predatory forays into spider webs, *Portia*'s vibratory signals on the silk of the resident's web often seem excessively variable over a short time span, and it has been suggested that the irregularity of these signals sustains the resident spider's interest and keeps it quietly out in the web, all the while hiding from the prey spider cues that might reveal to the prey spider that it is in peril of being attacked by a predator (Harland and Jackson 2004). An even more direct parallel to *Nagusta*'s and *S. repax*'s antennating is found in *S. bituberus*, as this araneophagic emesine also seems to calm prey by bouts of antennating prior to attacking (Wignall and Taylor 2008).

*Myrmarachne*, a myrmecomorphic salticid, was an especially interesting prey of *Nagusta* and *S. repax*. For *Myrmarachne*, myrmecomorphy probably functions largely in anti-predator defense, as there is strong evidence to suggest that all of the species in this large genus are Batesian mimics of ants (Edmunds 2006; Nelson and Jackson 2006b; Nelson et al. 2006). Like *Portia* (Nelson and Jackson 2006b), *Nagusta* and *S. repax* are both averse to ants, and yet both reduviids preyed on *Myrmarachne*. This suggests that, for *Nagusta* and *S. repax*, prey detection and identification depends primarily on cues in sensory modalities other than vision and that the sensory system of these reduviids is not fooled by *Myrmarachne*' s resemblance to ants (unlike that of *Portia*) (Nelson and Jackson 2006b).

Some reduviids have a special structure, known as the ‘cave organ’, on the pedicel of their antennae, and there is morphological evidence that these organs have a role in chemoreception (Weirauch 2008). However, Harpactorinae is one of the reduviid subfamilies that apparently have no cave organs. On the whole, surprisingly little is known about sensory systems of any reduviids, including even the medically significant hematophagous species that act as vectors for Chagas disease. The sensory systems of *Nagusta* and *S. repax* would be especially interesting topics for future research, as the behavior of these reduviids suggest remarkable prey-discrimination abilities.
